# The mechanistic role of gitogenin as a treatment for laryngeal cancer: a network pharmacology and experimental analysis

**DOI:** 10.3389/fphar.2025.1663323

**Published:** 2025-12-17

**Authors:** Zhuo Fu, Yue Guan, Zhangwei Hu

**Affiliations:** 1 School of Basic Medical Sciences, Inner Mongolia Minzu University, Tongliao, China; 2 Department of Otolaryngology, The First Affiliated Hospital, Sun Yat-sen University, Guangzhou, China

**Keywords:** gitogenin, laryngeal cancer, network pharmacology, PI3K-Akt pathway, therapeutic efficacy

## Abstract

**Objective:**

Laryngeal cancer (LC) is a significant and persistent therapeutic challenge worldwide. Gitogenin (GIT), a naturally occurring saponin, has demonstrated anticancer activity in lung cancer. However, its potential effects on LC remain unclear. This study aimed to elucidate the therapeutic efficacy of GIT and the mechanisms through which it acts against LC by integrating network pharmacology analysis with experimental validation.

**Methods:**

Potential targets of GIT were established using the PharmMapper database, while LC-associated genes were retrieved from the GeneCards database. Common targets between GIT and LC were determined, and the top 20 genes were chosen for protein-protein and gene-gene interaction (PPI and GGI) network construction. Functional enrichment analyses were conducted. Finally, *in vitro* experiments were performed to validate how GIT affects LC.

**Results:**

A total of 96 putative GIT targets for LC were identified. KEGG enrichment identified the PI3K-Akt axis a key regulatory mechanism. *In vitro* experiments provided confirmation that GIT inhibited LC cell proliferation, viability, migratory activity, and invasion ability by modulating PI3K-Akt signaling.

**Conclusion:**

Using network pharmacology and experimental validation, it was demonstrated that GIT exerts potent anticancer effects on LC by targeting the PI3K-Akt axis. The present findings suggest the potential of GIT for treating LC.

## 1 Introduction

Laryngeal cancer (LC) is a prevalent malignancy of the head and neck, contributing significantly to cancer-related morbidity and mortality (Anthony and Nicole, 2023). Approximately half of LC cases are diagnosed at advanced stages, leading to poor survival outcomes (Conor et al., 2016). Despite advancements in surgical techniques, chemotherapy, and radiotherapy, therapeutic efficacy remains limited due to functional impairment, drug resistance, and high recurrence rates (Randa et al., 2018; Michael et al., 2021). Consequently, the search for novel and effective treatment strategies for LC is of major importance.

Saponins, a class of amphiphilic compounds derived from plants, have been widely recognized for their diverse biological activities, including anticancer properties (Yu-Pu and Pi-Hui, 2020; Dongdong et al., 2025). Many saponins have demonstrated tumor-inhibitory effects across various cancers (Wei et al., 2024; Yang et al., 2023). Gitogenin (GIT), a naturally occurring saponin isolated from *Tribulus longipetalus* and *Hosta plantaginea*, has been shown to lower mean arterial pressure in rats by promoting endothelial vasodilation and inhibiting atrial contraction (Imran et al., 2021). Recent studies, such as those by Liu et al., have revealed that GIT suppresses lung cancer progression by inducing apoptosis and autophagy, suggesting its potential as an anticancer agent (Ting et al., 2022). However, its role in LC remains unexplored.

**FIGURE 1:**
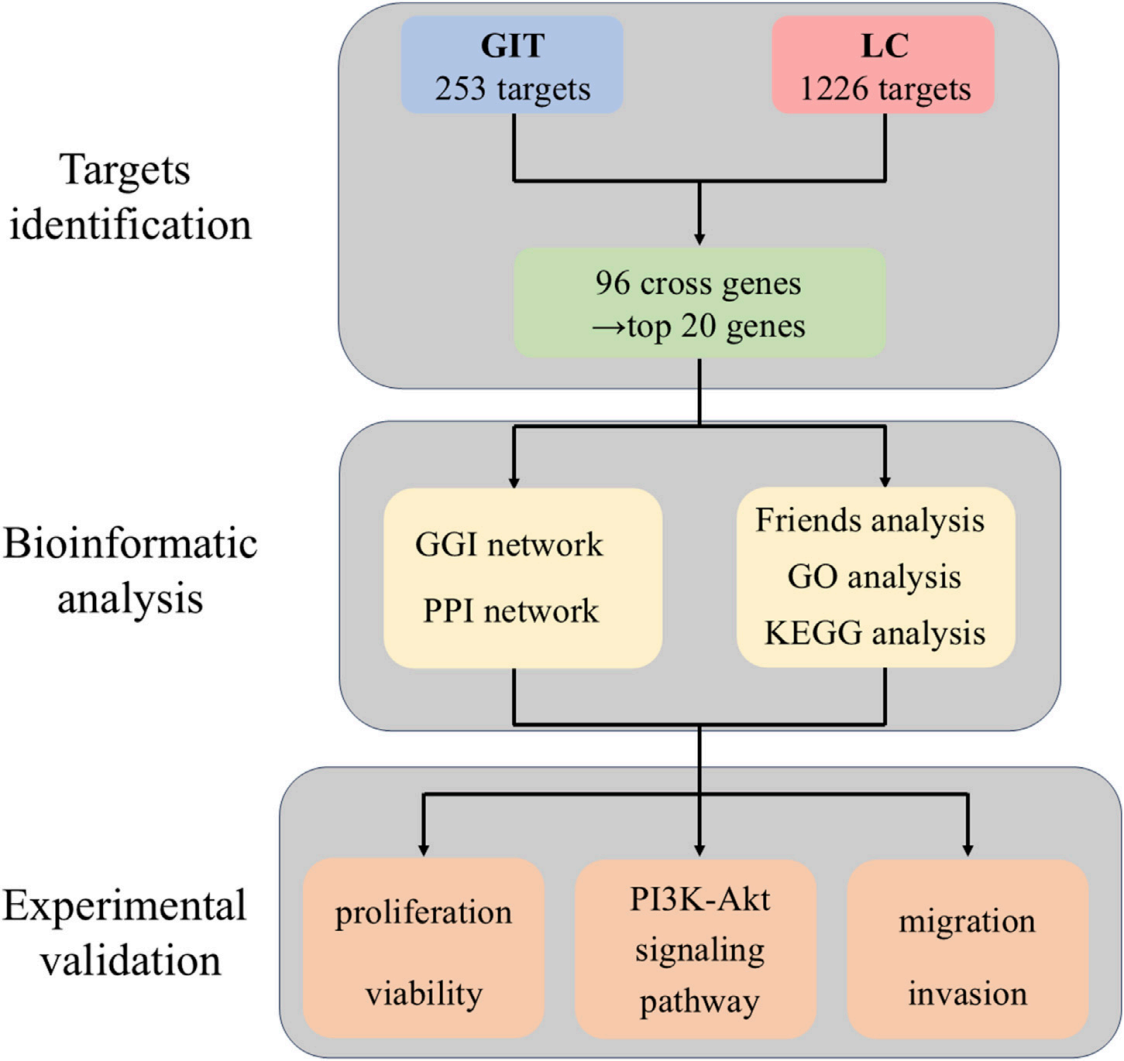
Overview of the use of gitogenin in laryngeal cancer.

Given the complexity of drug mechanisms involving multiple targets and pathways, network pharmacology provides a valuable approach to elucidate drug-disease interactions (Cristian et al., 2021; Muthuramalingam et al., 2023). In head and neck cancer, network pharmacology has improved our understanding of the tumor targets linked to bioactives of plants (Muthuramalingam et al., 2024). Gao et al., found that the traditional Chinese medicine decoction Baiying Qinghou performed therapeutic effect against LC by regulating TP53, EGFR, NOS3 and IL1B gene targets and PI3K-AKT signaling pathway through network pharmacology analysis (Gao et al., 2021). This study employed network pharmacology analysis and *in vitro* approaches to investigate the molecular mechanisms of GIT against LC. Potential targets were predicted through bioinformatics analyses, followed by experimental validation of therapeutic effects and signaling pathways in LC cells. The study workflow is illustrated in Figure 1.

## 2 Materials and methods

### 2.1 Identification of GIT-associated targets and LC-related genes

The chemical structure and SMILES format of GIT were obtained from PubChem (https://pubchem.ncbi.nlm.nih.gov/). Possible GIT target predictions were made with PharmMapper (http://www.lilab-ecust.cn/pharmmapper/), with conversion to protein symbols with UniProt (https://www.uniprot.org/). LC-associated genes were identified in GeneCards (https://www.genecards.org/) by searching for “laryngeal cancer,” with a correlation score threshold of ≥10. Overlapping targets between GIT and LC were determined with Draw Venn Diagram tool (https://bioinformatics.psb.ugent.be/webtools/Venn/), and the expression of the shared targets in LC tissues was assessed using UALCAN (http://ualcan.path.uab.edu/) (Accessed date: 2024.11.29).

### 2.2 Protein-protein and gene-gene interaction (PPI and GGI) network construction

The top 20 common targets, ranked by relevance scores, were selected for further analysis (Supplementary Table S1; Table 1). These core genes were uploaded to the STRING database (https://string-db.org/) to construct the PPI network. Additionally, the GeneMANIA database (http://www.genemania.org/) was used to develop the GGI network (Accessed date: 2024.12.01).

**TABLE 1:** Top 20 cross genes of GIT for LC.

Gene symbol	Relevance score
EGFR	136.4335632
MET	109.8416214
KIT	86.07278442
ESR1	70.32738495
AR	64.95728302
FGFR2	63.36435318
MDM2	53.8009491
SRC	50.16234589
FGFR1	49.73897171
ERBB4	47.17902756
MAP2K1	45.61016464
AURKA	43.36730194
MAPK1	41.86813736
DHFR	41.26192474
GSTM1	39.16648102
KDR	38.68711472
IL2	38.52807236
PTPN11	37.96383667
MMP2	37.21245575
PIK3CG	36.27074814

### 2.3 Functional enrichment analyses

To evaluate the functional relevance of key genes, the top 20 genes were analyzed using the R package GOSemSim [2.22.0]. The 96 overlapping genes and these top 20 genes were then subjected to GO and KEGG analyses were performed using DAVID (https://david.ncifcrf.gov/). The R package ggplot2 [3.4.4] was employed to generate visualization charts for the top three enriched terms in each category (Accessed date: 2024.12.01).

### 2.4 Chemicals, reagents, and cell culture

GIT (CAS No.: 511-96-6) was purchased from MedChemExpress (MCE, NJ, United States). Other reagents, including DMSO, CCK-8 kit, staining kits, paraformaldehyde, propidium iodide (PI), TRI reagent, and ECL chemiluminescence reagent, were obtained from Biosharp (Hefei, China). Matrigel, cell lysis buffer, protease and phosphatase inhibitor cocktails, and the BCA protein assay kit were sourced from Beyotime (Shanghai, China). Antibodies were supplied by Affinity Biosciences (Liyang, China).

The Hep-2 and TU212 human LC cell line was obtained from iCell Bioscience (Shanghai, China). Cells were grown in RPMI 1640 (Gibco, MA, United States) containing 10% fetal bovine serum (CLARK, VA, United States) at 37 °C in a humidified 5% CO_2_ atmosphere.

### 2.5 Cell viability analyses

Cells were inoculated in 96-well plates (1 × 10^4^/well) for 24 h, and then treated with various GIT doses for 24 h. After subsequently adding CCK-8 solution, a 2-h incubation was performed, and absorbance was quantified at 450 nm using a microplate reader (Tecan, Männedorf, Switzerland).

### 2.6 Colony formation assay

Cells (1,000/well) were inoculated in 6-well plates and grown for 48 h. Cells were treated using different concentrations of GIT for 24 h, followed by further incubation with media replenishment every 3 days until colony appearance. Cells were fixed with 4% paraformaldehyde, stained using a commercial staining kit, and photographed via camera.

### 2.7 Immunofluorescence

Cells were added to glass-bottom dishes and followed by GIT treatment for 24 h, after which the cells were rinsed with PBS and stained with PI solution as directed. Brightfield and fluorescence images were captured using a fluorescence microscope (Olympus, Tokyo, Japan).

### 2.8 Wound healing assay

Cells were seeded into 6-well plates and cultured until confluent. A linear scratch was created with a sterile pipette tip, and the cells were washed using PBS to eliminate debris. Subsequently, the cells were treated using GIT solution and incubated for 24 h. The wounds were imaged at 0 and 24 h using a microscope, and migration was assessed by measuring the relative size of the wounds at 24 h compared with at 0 h post scratch with Fiji (ImageJ distribution, WI, United States).

### 2.9 Transwell assay

Cells were suspended using serum-free medium containing varying concentrations of GIT and added to the upper chamber of Matrigel-coated (1:8 in PBS) Transwell inserts. After filling the lower compartment with 10% FBS-containing medium, plates were incubated for 24 h, and PBS was used to wash cells followed by 4% paraformaldehyde fixation, after which a staining kit was used for staining. Migratory cells (cells attached to the underside of the Transwell chambers) were visualized using a microscope (Olympus, Tokyo, Japan) and counted.

### 2.10 Kaplan-Meier Plotter analyses

The prognostic value of genes associated with the PI3K-Akt axis was evaluated with Kaplan-Meier Plotter (http://kmplot.com/analysis/). A cohort of 499 patients was stratified into high- and low-expression groups for PIK3CA and AKT1 based on median gene expression levels. Overall survival (OS) was compared using Kaplan-Meier survival curves.

### 2.11 qRT-PCR

Cells were grown in 12-well plates followed by treatment with GIT for 24 h. Total RNA was extracted with the TRI reagent and processed into cDNA with the HiScript III RT SuperMix kit (Vazyme, Nanjing, China). qRT-PCR was conducted using SYBR Green Master Mix (ThermoFisher, MA, United States) on an ABI StepOne Plus system (ABI, CA, United States). Primer sequences: PIK3CA (F: 5′-TCT​GTC​TCC​TCT​AAA​CCC​TG-3′, R: 5′-TTC​TCC​CAA​TTC​AAC​CAC-3′), AKT1 (F: 5′-GCG​AGC​TGT​TCT​TCC​ACC-3′, R: 5′-CCT​TGT​CCA​GCA​TGA​GGT​T-3′), and β-Actin (F: 5′-TTC​AAC​ACC​CCA​GCC​ATG-3′, R: 5′-CTC​GTA​GAT​GGG​CAC​AGT-3′). β-Actin represented the internal reference, with relative gene expression determined using the 2^−ΔΔCT^ method.

### 2.12 Western blotting

Cells in 6-well plates were treated for 24 h using various GIT doses, followed by lysis with a lysis buffer containing protease and phosphatase inhibitor cocktails. After quantitification by BCA assays, equal protein amounts were separated via SDS-PAGE and transferred onto PVDF membranes. Following a 1-h block, the blots were treated overnight at 4 °C with primary antibodies against PI3K, p-PI3K, AKT1, p-AKT1, and GAPDH. Secondary antibodies were then used to probe blots for 1 h at room temperature. Protein band detection was performed with an ECL chemiluminescence reagent and visualized with a UVP ChemSolo Auto system (Analytic Jena, Jena, Germany).

### 2.13 Statistical analyses

GraphPad Prism 8 (GraphPad Software, CA, United States) was employed for all analyses. Data are given as means ± SD and compared with one-way ANOVAs. P < 0.05 was deemed significant.

## 3 Results

### 3.1 Identification of GIT targets in LC

**FIGURE 2:**
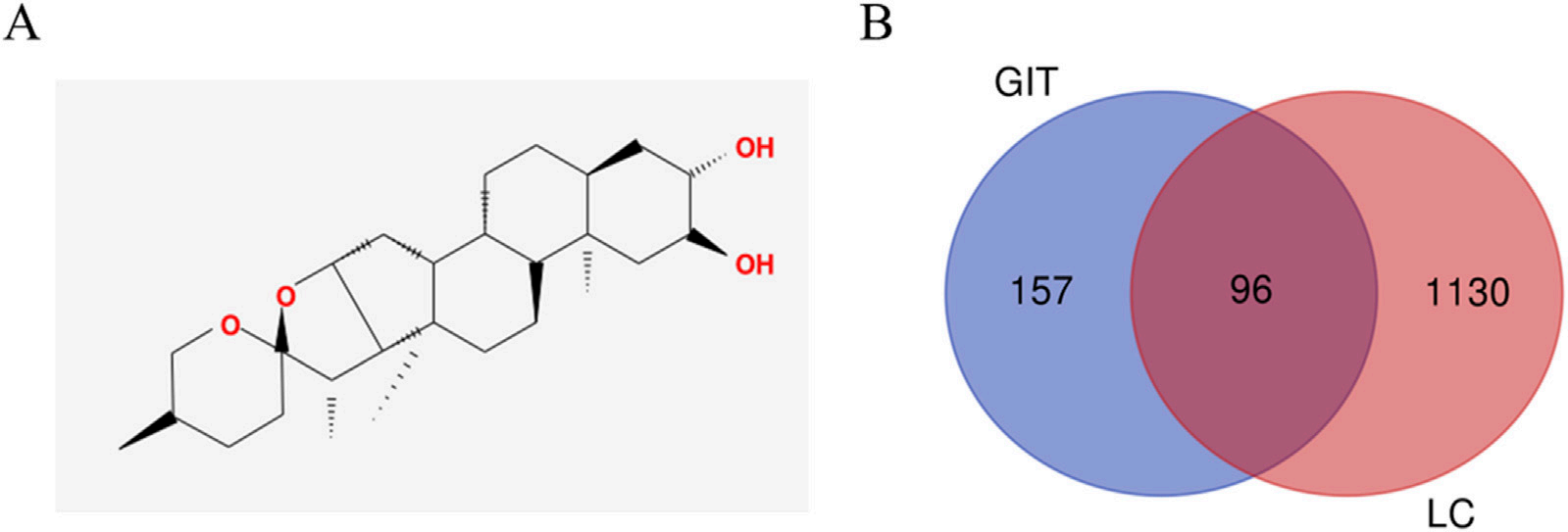
Targets of gitogenin (GIT) in treating laryngeal cancer (LC). **(A)** The chemical structure of GIT. **(B)** Venn diagram showing the 96 target genes overlapping between GIT and LC.

**FIGURE 3:**
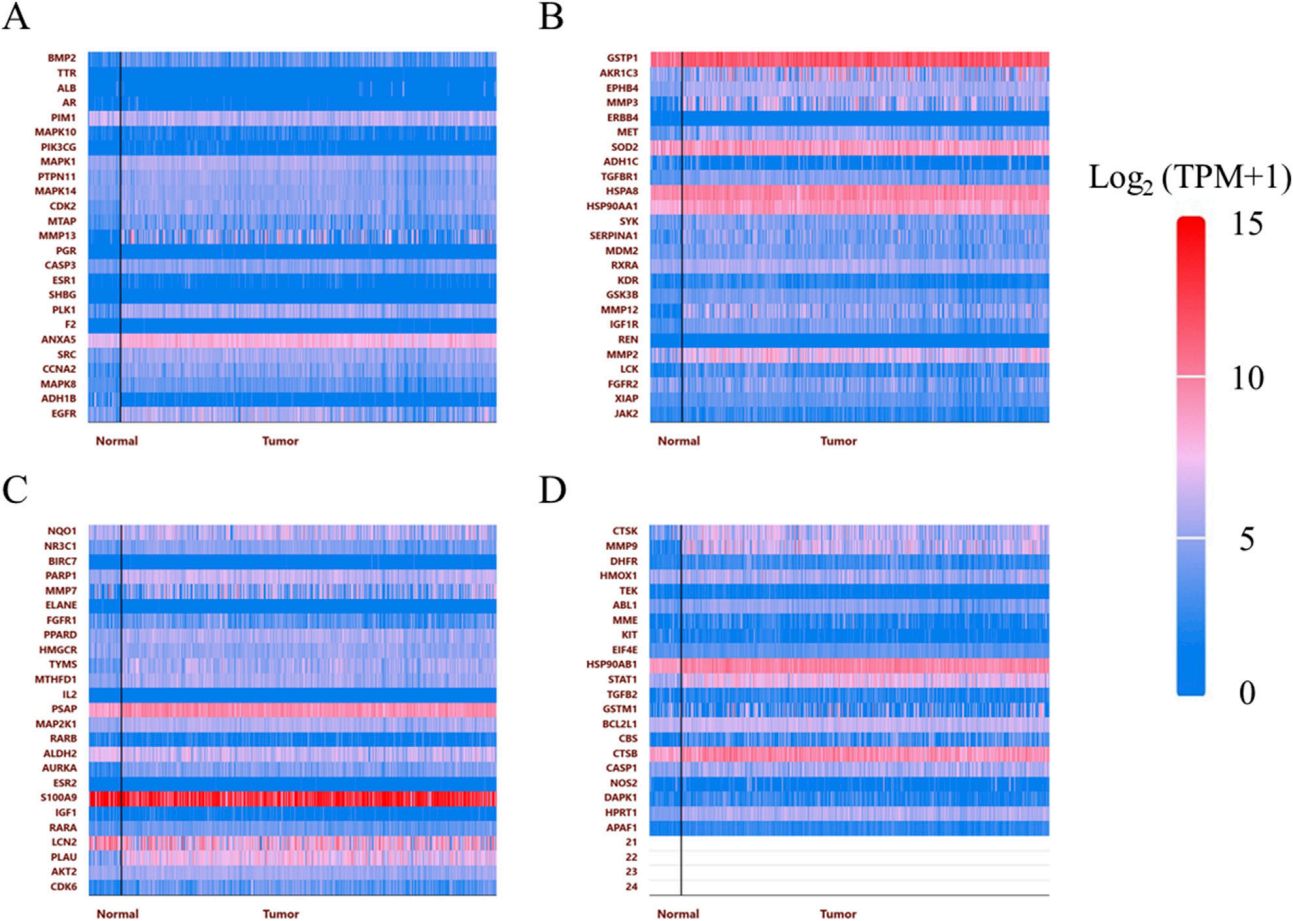
Target gene expression. **(A–D)** Expression analyses of genes overlapping between GIT and LC in head and neck cancer and normal tissue samples.

The chemical structure of GIT is presented in Figure 2A. In total, 253 GIT-associated targets were retrieved from the PharmMapper database, while 1,226 LC-related genes were identified from the GeneCards database. By analyzing their intersection using the Draw Venn Diagram tool, 96 potential GIT targets for LC were identified (Figure 2B). The expression levels of these 96 overlapping genes in head and neck cancer tissues relative to normal tissues were assessed using the UALCAN database (Figures 3A–D).

### 3.2 PPI and GGI network development

**FIGURE 4:**
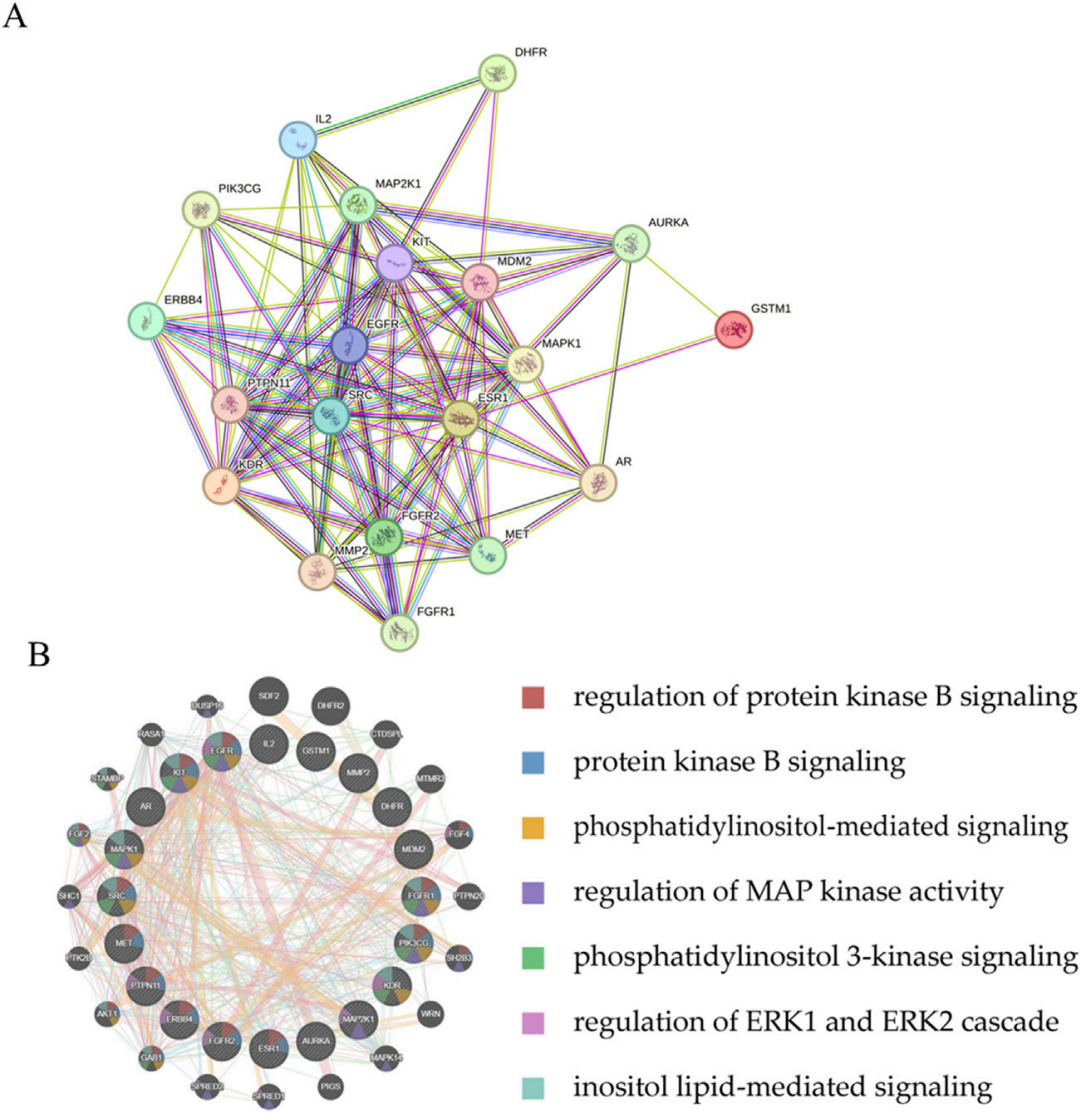
Core gene network diagram. **(A)** PPI network of the 20 top genes. **(B)** GGI network of top target genes.

The top 20 overlapping genes, ranked by relevance score, were used for constructing a PPI network in STRING, with the network comprising 20 nodes and 109 edges (Figure 4A). The same genes were additionally analyzed in the GeneMANIA database to generate the GGI network (Figure 4B).

### 3.3 Functional enrichment analyses

**FIGURE 5:**
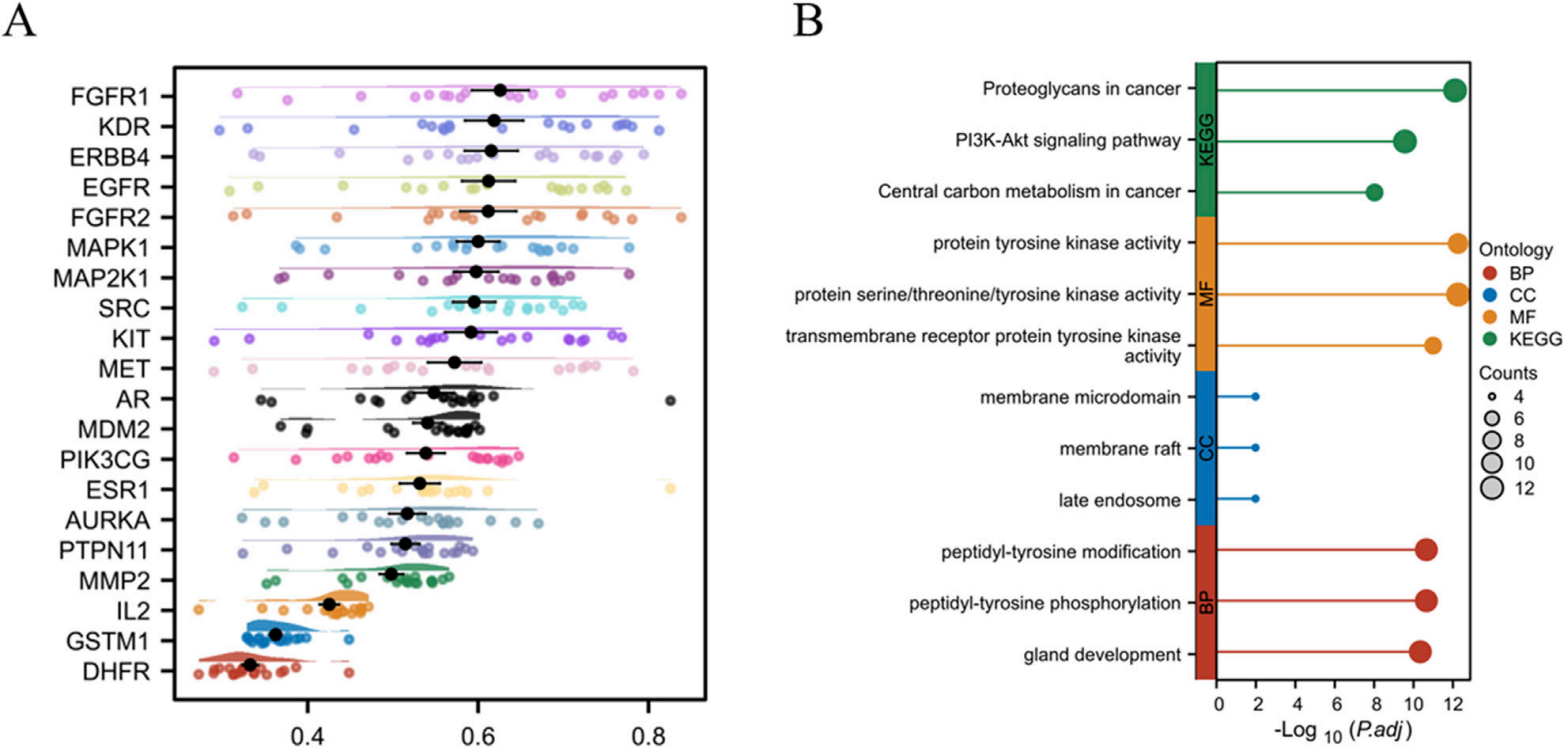
Core gene enrichment analysis. **(A)** Friends analysis of core genes. **(B)** GO and KEGG enrichment analyses of top genes.

A Friends analysis of the top 20 overlapping genes revealed that FGFR1 exhibited the highest connectivity, suggesting its potential role in LC treatment (Figure 5A). GO annotation analysis identified 725 biological processes (BPs), 10 cellular components (CCs), and 69 molecular functions (MFs) (P < 0.05). The top three enriched terms based on P-values are shown in Figure 5B. The most significant BPs included peptidyl-tyrosine modification, peptidyl-tyrosine phosphorylation, and gland development. The top CCs included membrane microdomains, membrane rafts, and late endosomes, while the most significant MFs involved transmembrane receptor protein tyrosine kinase activity, protein tyrosine kinase activity, and protein serine/threonine/tyrosine kinase activity. KEGG analyses revealed pathways associated with proteoglycans in cancer, the PI3K-Akt axis, and central carbon metabolism in cancer as the three most significantly enriched pathways. Similar KEGG pathway results were obtained when analyzing all 96 overlapping genes, confirming the importance of the PI3K-Akt axis in LC (Supplementary Figure S1). As this pathway is known to be linked to tumor progression and potential relevance in LC treatment, the effects of GIT on the axis were further investigated.

### 3.4 GIT inhibits LC cell proliferation and viability

**FIGURE 6:**
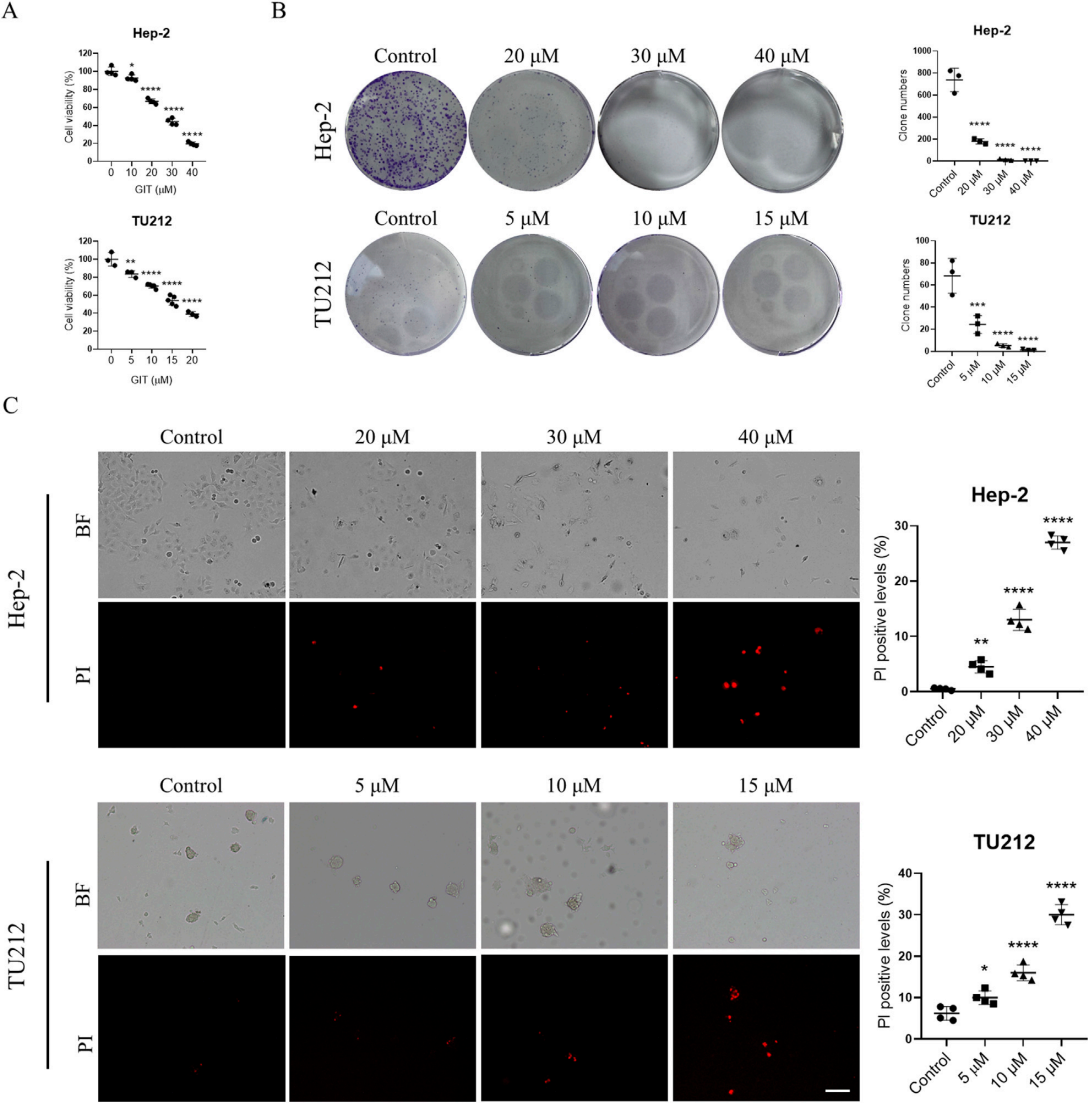
Gitogenin (GIT) inhibits laryngeal cancer (LC) cell proliferation and viability. **(A)** CCK-8-based analysis of cell viability. **(B)** Colony formation assay-based analysis of cell proliferation. **(C)** PI staining-based analysis of cell apoptosis. BF, bright field. Data are presented as the means ± standard error of the mean. Scale bar, 100 μm *p < 0.05, **p < 0.01, ***p < 0.001, ****p < 0.0001.

To evaluate the impact of GIT on LC progression, Hep-2 and TU212 cells were treated using varying doses of GIT. As presented in Figure 6A, CCK-8 assays demonstrated that GIT significantly decreased LC cell viability in a dose-dependent fashion. Accordingly, 20, 30, and 40 μM GIT concentrations were selected for subsequent Hep-2 cell experiments, while 5, 10 and 15 μM GIT concentrations were selected for TU212 cell experiments. Consistently, colony formation assays demonstrated a significant reduction in the proliferative capacity of both cells treated with GIT relative to controls (Figure 6B). Additionally, PI staining indicated a dose-dependent increase in apoptosis following GIT treatment, confirming its cytotoxic effects on LC cells (Figure 6C).

### 3.5 GIT suppresses LC cell migration and invasion

**FIGURE 7:**
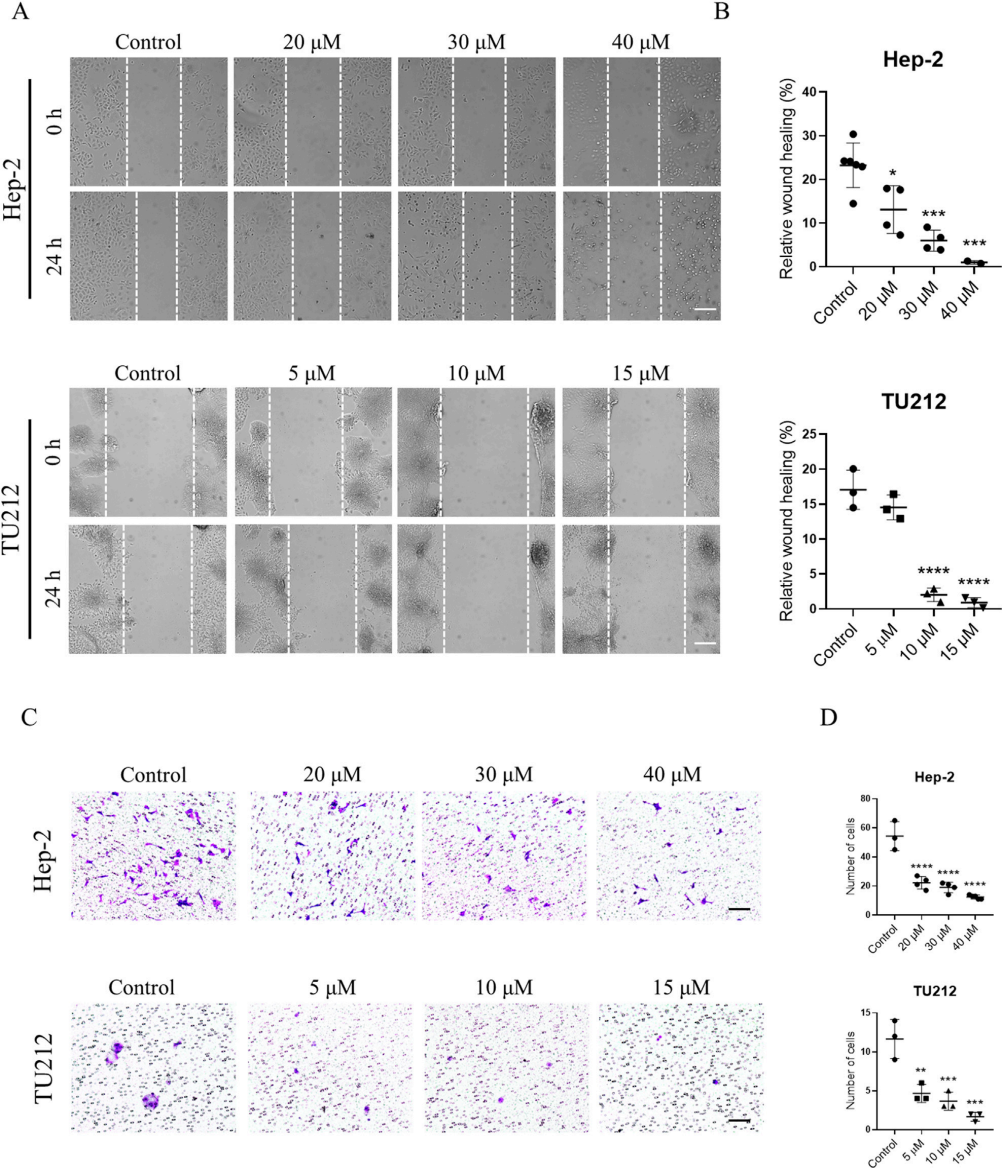
Gitogenin (GIT) inhibits laryngeal cancer (LC) cell migration and invasion. **(A,B)** Wound healing assay-based analysis of cell migration. **(C,D)** Transwell assay-based analysis of cell invasion. Data are presented as the means ± standard error of the mean. Scale bar, 100 μm *p < 0.05, **p < 0.01, ***p < 0.001, ****p < 0.0001.

Cell migration and invasion are central to tumor metastasis. The results of a wound healing assay demonstrated that GIT treatment markedly and dose-dependently reduced the migratory capacity of Hep-2 and TU212 cells (Figures 7A,B). Similarly, Transwell invasion assays showed a substantial reduction in invasive cell numbers following GIT treatment compared to the control group (Figures 7C,D). These findings show that GIT can inhibit LC cell migration and invasion effectively, further supporting its therapeutic potential.

### 3.6 PI3K-Akt expression is enhanced in LC and correlates with poor prognosis

**FIGURE 8:**
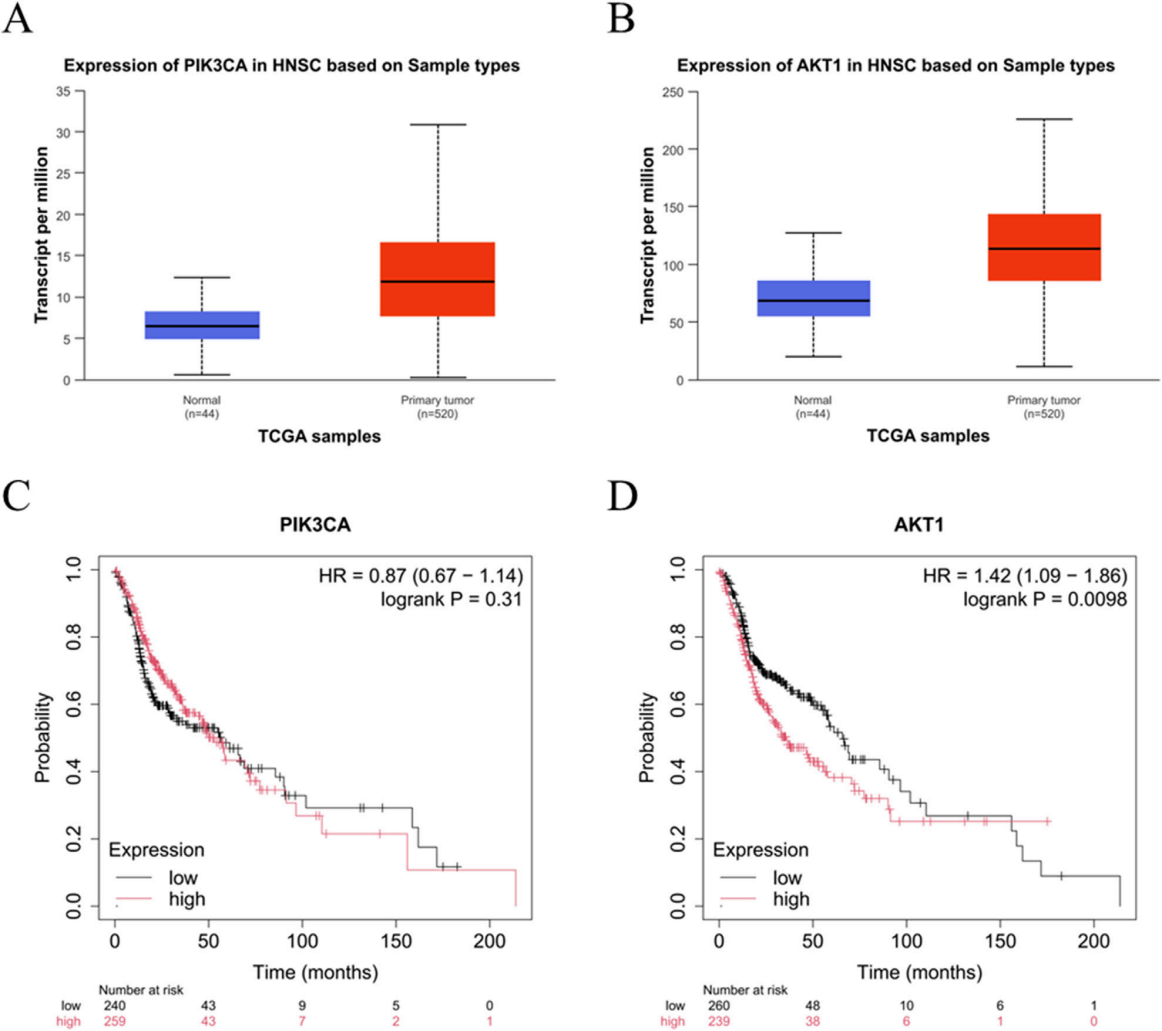
PI3K-Akt signaling pathway expression and survival analyses in head and neck cancer. **(A,B)** UALCAN database analyses of PIK3CA **(A)** and AKT1 **(B)** gene expression in normal and tumor tissues. **(C,D)** Kaplan-Meier plotter database analyses of the prognostic utility of PIK3CA **(C)** and AKT1 **(D)** in head and neck cancer.

The preceding results revealed that GIT effectively inhibits LC cell proliferation, viability, migration, and invasion. Network pharmacology showed that the close involvement of the PI3K-Akt axis in GIT-mediated LC treatment. To determine whether GIT exerts its anti-cancer effects through this pathway, we conducted both database analyses and experimental verification. Analysis using the UALCAN database revealed that the key genes PIK3CA and Akt1 were significantly overexpressed in head and neck squamous cell carcinoma tumors relative to normal tissues (Figures 8A,B). Furthermore, the expression of PIK3CA and Akt1 in head and neck cancer based on nodal metastasis status was also investigated (Supplementary Figure S2). Additionally, Kaplan-Meier analyses demonstrated that elevated AKT1 expression was related to poorer prognosis (Figures 8C,D). These results suggest the close involvement of the PI3K-Akt axis in LC progression and its potential for therapeutic intervention.

### 3.7 GIT inhibits PI3K-Akt signaling in LC cells

**FIGURE 9:**
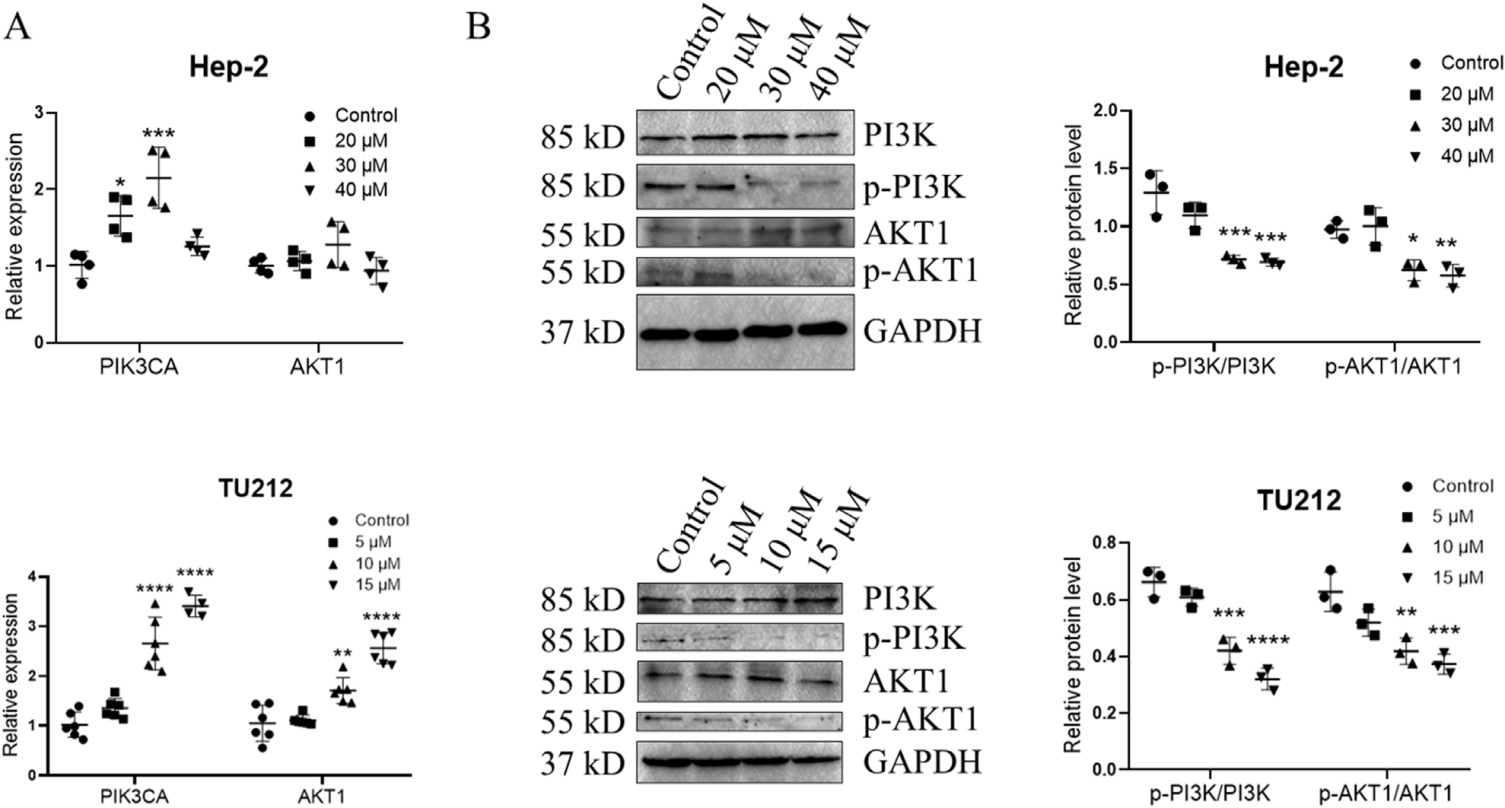
Gitogenin (GIT) inhibits PI3K-Akt pathway activation. qRT-PCR was used to analyze the relative expression of PIK3CA and AKT1 **(A)**. **(B)** Western blotting analysis of PI3K, p-PI3K, AKT1 and p-AKT1. Data are presented as the means ± standard error of the mean. *p < 0.05, ***p < 0.001.

To further evaluate this hypothesis, qRT-PCR and Western blotting were conducted. qRT-PCR analysis showed that treatment with medium and high concentration GIT led to a slight upregulation of PIK3CA and AKT1 expression in Hep-2 cells and TU212 cells (Figure 9A). Western blotting further demonstrated that GIT markedly reduced PI3K and Akt protein phosphorylation (Figure 9B), indicating that GIT effectively inhibits the activation of the PI3K-Akt axis in LC cells. These combined bioinformatics and experimental findings confirm that the antitumor effects of GIT in LC are driven through the suppression of this pathway.

## 4 Discussion

LC remains among the most prevalent tumors of the respiratory system, with an estimated 12,650 new cases and 3,880 deaths projected in 2024 (Rebecca et al., 2024). Given the severe impact of LC on public health, exploring novel and effective treatment approaches is urgently required. Traditional Chinese medicine, known for its multi-target and multi-pathway approach, has shown promise in cancer treatment (Zhang et al., 2021; Zimei et al., 2020). GIT, a natural saponin derived from plants, has demonstrated anti-cancer properties in lung cancer. However, its role in LC and the underlying mechanisms remain largely unexplored. Here, the integration of network pharmacology with experimental approaches was conducted to clarify the anti-cancer efficacy of GIT in LC and to identify its potential molecular mechanisms.

Network pharmacology analysis was employed for predicting GIT-relate targets and the mechanisms through which it combats LC. A total of 96 overlapping targets were detected from among 253 putative GIT targets and 1,226 LC-related genes. The expression of these genes was compared between normal and LC tissues. The top 20 cross-targets were subsequently selected for the construction of PPI and GGI networks, revealing their involvement in key signaling pathways such as protein kinase B signaling, phosphatidylinositol-mediated signaling, MAP kinase regulation, PI3K signaling, ERK1/ERK2 cascade regulation, and inositol lipid-mediated signaling. Further enrichment analyses, including Friends, GO and KEGG analyses, unveiled the PI3K-Akt axis as critically important.

Through *in vitro* experimentation, this study confirmed the therapeutic effects of GIT on LC cells. The present findings indicated that GIT treatment significantly reduced LC cell proliferation and viability while inducing apoptosis. This fits with research by Liu et al. on lung cancer cells, further supporting the anti-cancer function of GIT (Ting et al., 2022). Additionally, GIT was found to suppress LC cell migration and invasion, indicating its potential role in inhibiting tumor progression and metastasis.

The highly conserved PI3K-Akt signal transduction pathway is associated with cellular survival, growth, and proliferation (Le et al., 2021). Recent studies have thoroughly interrogated the role of this pathway in various cancers, including breast, colon, gastric, and pancreatic cancer (Carla and Henrik, 2023; Peisi et al., 2023; Ayda et al., 2021; Ruiyuan et al., 2023). Our network pharmacology analysis predicted that the PI3K-Akt axis might be a key mechanism underlying GIT’s anti-LC effects. Furthermore, bioinformatics analysis revealed that PIK3CA and AKT1, two major functional genes within this pathway, were strongly expressed in LC tissues and linked to poor prognosis. Cellular experiments further confirmed that GIT treatment led to a reduction in PI3K, p-PI3K, AKT, and p-AKT levels in LC cells, supporting the pivotal function of the PI3K-Akt axis in GIT-mediated LC treatment.

## 5 Conclusion

**FIGURE 10:**
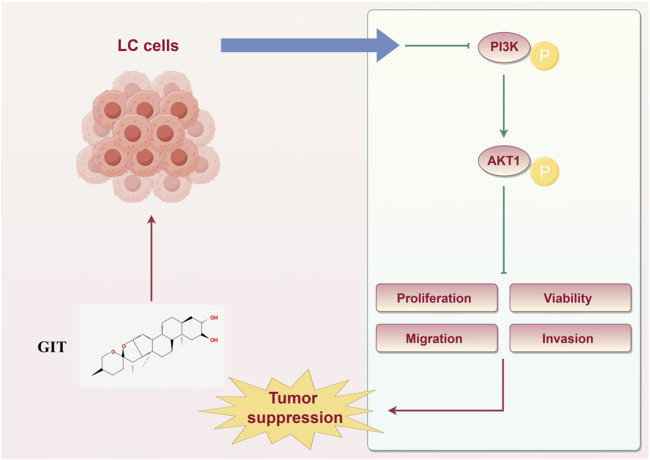
Schematic illustration of the therapeutic effects of gitogenin on laryngeal cancer.

In summary, this study provides a comprehensive overview of the effects of GIT in LC treatment and the associated mechanisms based on network pharmacology analyses and corresponding experimental validation (Figure 10). These findings demonstrate that GIT exerts significant anti-cancer effects in LC, primarily through the suppression of the PI3K-Akt signaling pathway. Collectively, these results provide strong evidence that GIT may be a promising therapeutic strategy for LC. However, as this study was limited to bioinformatics analysis and cellular experiments, further validation through *in vivo* animal studies is necessary to fully establish the therapeutic promise of GIT in LC.

## Data Availability

The raw data supporting the conclusions of this article will be made available by the authors, without undue reservation.
