# Supplementary material for: The mechanistic role of gitogenin as a treatment for laryngeal cancer: a network pharmacology and experimental analysis
Source: Front Pharmacol. 2025 Dec 17;16:1663323. doi: 10.3389/fphar.2025.1663323 (PMC12753904; doi:10.3389/fphar.2025.1663323)
Supplement: Supplementary file 1 [file DataSheet1.docx]

Supplementary Material

# Supplementary Figures and Tables

## Supplementary Figures


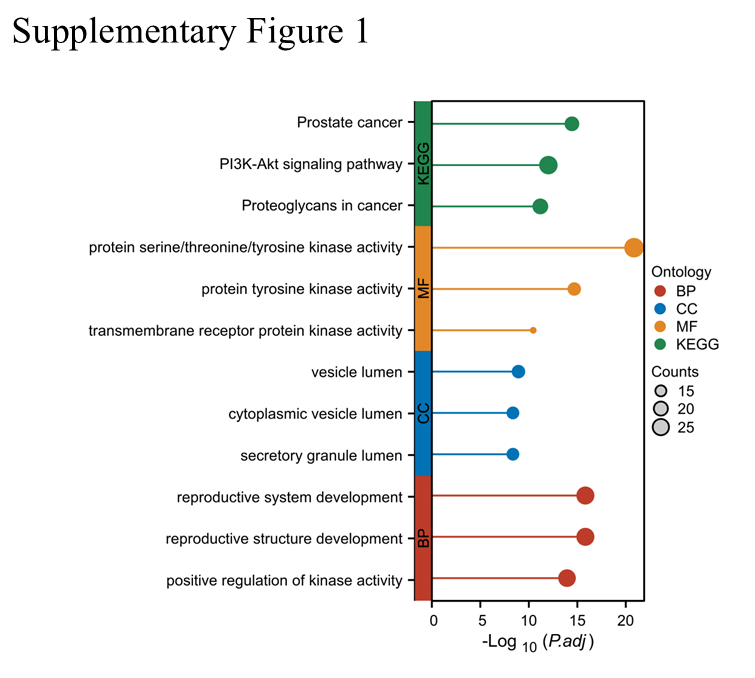


**Supplementary Figure 1.** GO and KEGG analysis of total 96 overlapping genes.


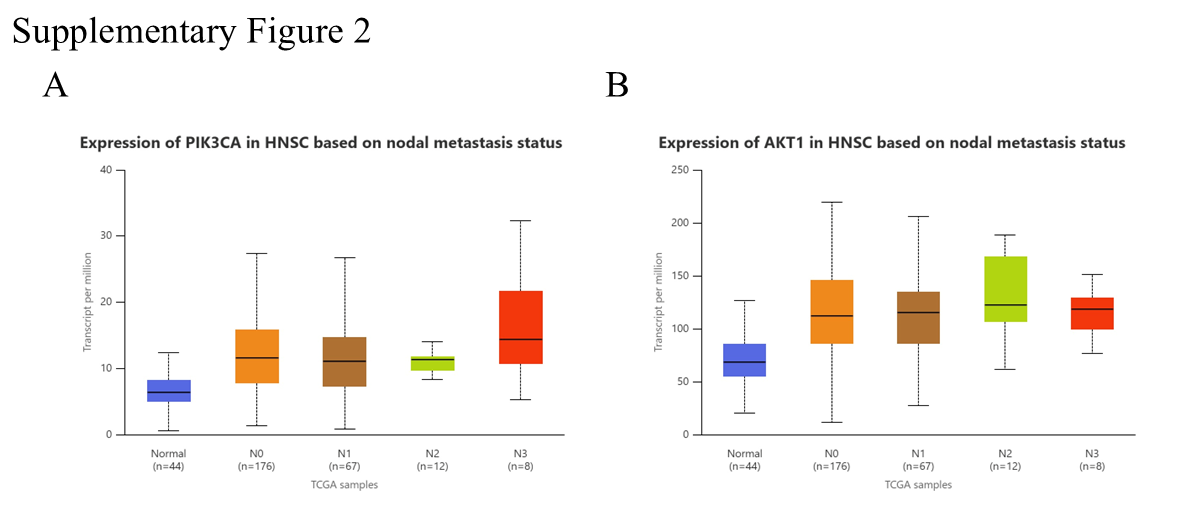


**Supplementary Figure 2.** PI3K-Akt signaling pathway expression in metastatic head and neck cancer. UALCAN database analyses of PIK3CA (A) and AKT1 (B) gene expression in head and neck cancer based on nodal metastasis status.

## Supplementary Tables

**Supplementary Table 1.** Potential genes of GIT against LC

| **Gene Symbol** | **Relevance score** |
| --- | --- |
| EGFR | 136.4335632 |
| MET | 109.8416214 |
| KIT | 86.07278442 |
| ESR1 | 70.32738495 |
| AR | 64.95728302 |
| FGFR2 | 63.36435318 |
| MDM2 | 53.8009491 |
| SRC | 50.16234589 |
| FGFR1 | 49.73897171 |
| ERBB4 | 47.17902756 |
| MAP2K1 | 45.61016464 |
| AURKA | 43.36730194 |
| MAPK1 | 41.86813736 |
| DHFR | 41.26192474 |
| GSTM1 | 39.16648102 |
| KDR | 38.68711472 |
| IL2 | 38.52807236 |
| PTPN11 | 37.96383667 |
| MMP2 | 37.21245575 |
| PIK3CG | 36.27074814 |
| MMP9 | 35.04437637 |
| GSTP1 | 34.24658203 |
| IGF1R | 33.17932892 |
| CDK2 | 32.80148315 |
| CASP3 | 32.03458786 |
| SOD2 | 31.763237 |
| CDK6 | 31.24964142 |
| JAK2 | 30.96692848 |
| RARB | 30.63274956 |
| TYMS | 30.09782028 |
| IGF1 | 29.72334099 |
| BCL2L1 | 28.18684769 |
| TGFBR1 | 27.94408035 |
| PGR | 27.74307823 |
| AKT2 | 27.24709702 |
| ABL1 | 27.24192429 |
| ESR2 | 26.2838726 |
| PARP1 | 25.74199486 |
| GSK3B | 25.45711708 |
| ALB | 24.20785141 |
| PLAU | 23.82192039 |
| HSP90AA1 | 23.53821945 |
| NQO1 | 23.31283569 |
| STAT1 | 22.54141617 |
| MMP7 | 22.17216682 |
| EPHB4 | 22.14269638 |
| XIAP | 21.76075172 |
| CBS | 21.74988937 |
| MAPK10 | 21.18540573 |
| NOS2 | 20.81734085 |
| RARA | 20.51327705 |
| PLK1 | 20.33307457 |
| MAPK14 | 20.22589874 |
| MAPK8 | 19.98208427 |
| RXRA | 19.76661301 |
| CCNA2 | 19.35222435 |
| BMP2 | 18.82414246 |
| TGFB2 | 18.37266541 |
| HMOX1 | 18.21001244 |
| ALDH2 | 17.73308945 |
| TEK | 17.63022232 |
| EIF4E | 17.49224472 |
| MME | 17.35321236 |
| DAPK1 | 17.34251595 |
| ANXA5 | 17.2165699 |
| PIM1 | 17.12519836 |
| MMP3 | 16.84663391 |
| ELANE | 16.29921341 |
| CTSB | 15.52966499 |
| MMP13 | 15.19195747 |
| SYK | 14.37602425 |
| HSP90AB1 | 14.28314018 |
| SHBG | 14.25245571 |
| SERPINA1 | 14.13037682 |
| TTR | 14.00499725 |
| APAF1 | 13.98139858 |
| LCN2 | 13.49282169 |
| CASP1 | 13.21614742 |
| NR3C1 | 13.13366795 |
| MTAP | 12.8487463 |
| HSPA8 | 12.7370863 |
| F2 | 12.52834511 |
| REN | 12.20242405 |
| BIRC7 | 11.94829082 |
| HMGCR | 11.81535721 |
| PPARD | 11.76156902 |
| S100A9 | 11.73703384 |
| AKR1C3 | 11.50150776 |
| MTHFD1 | 11.49318314 |
| ADH1B | 10.98959351 |
| LCK | 10.95137596 |
| ADH1C | 10.88489723 |
| MMP12 | 10.62097168 |
| PSAP | 10.45171738 |
| CTSK | 10.11692238 |
| HPRT1 | 10.10865593 |
